# Pain experiences of Tibetan children with hematologic malignancies in China: A descriptive phenomenological study

**DOI:** 10.1371/journal.pone.0360911

**Published:** 2026-10-16

**Authors:** Yi Chen, Yu Liu, Juan Zhou, Huarong Pu, Jinglan Zhang

**Affiliations:** 1 Cancer Center, Sichuan Provincial People’s Hospital, School of Medicine, University of Electronic Science and Technology of China, Chengdu, Sichuan, China; 2 Department of Oncology, Sichuan Provincial People’s Hospital, University of Electronic Science and Technology of China, Chengdu, China; 3 Department of Pediatric Hematology and Oncology, Sichuan Provincial People’s Hospital, University of Electronic Science and Technology of China, Chengdu, China; 4 Department of Nursing, Sichuan Provincial People’s Hospital, School of Medicine, University of Electronic Science and Technology of China, Chengdu, China; The Hong Kong Polytechnic University, HONG KONG

## Abstract

**Background:**

Pain is common in children with hematologic malignancies. It can be highly distressing and can shape how children feel and function during hospitalization. Children’s pain experiences are influenced not only by disease and treatment but also by developmental level, family context, language, and culture. However, little is known about how Tibetan children with hematologic malignancies experience and communicate pain in clinical settings.

**Aim:**

We aimed to explore the pain experiences of Tibetan children with hematologic malignancies and to inform culturally responsive pain assessment and nursing care.

**Methods:**

We used a descriptive phenomenological design. From February to March 2026, we purposively recruited 15 Tibetan children with hematologic malignancies from a pediatric hematology-oncology ward in Sichuan, China. Data were collected through play-based, semi-structured face-to-face interviews with children and analyzed using an adapted version of Colaizzi’s phenomenological method.

**Results:**

Four themes were identified: (1) cultural and spiritual interpretations of pain, including silent endurance and religious or traditional coping; (2) linguistic and communicative constraints in pain expression, including limited Mandarin vocabulary and indirect expression of discomfort; (3) family-centered dimensions of pain experience, including emotional security through family presence, relational continuity through family accompaniment, and familiar family-based comfort practices; and (4) hospitalization-related disruptions to everyday social life, including disrupted schooling and peer contact and concerns about illness disclosure and social isolation.

**Conclusions:**

The findings suggest that culturally responsive pain assessment may benefit from child-centered communication, attention to indirect and nonverbal expressions of pain, and recognition of the supportive role of family relationships and familiar comfort practices.

## 1. Introduction

Hematologic malignancies, particularly leukemia and lymphoma, account for a substantial proportion of childhood cancers worldwide [1]. Pain is frequently experienced during cancer treatment and may arise from the disease itself, treatment-related complications, surgery, or repeated diagnostic and therapeutic procedures [2,3]. Pediatric cancer pain is multidimensional; it may coexist with anxiety and sleep disturbance and can interfere with daily functioning and quality of life [4–6]. Accordingly, contemporary pediatric pain guidance emphasizes developmentally appropriate and systematic assessment, together with timely multimodal management and the prevention of procedure-related pain and distress [7,8]. In pediatric oncology, child-centered communication is particularly important because children’s communication needs and participation preferences vary according to age, developmental level, emotional state, and clinical context [9,10]. Children may communicate pain verbally, nonverbally, symbolically, or through subtle behavioral changes; failure to recognize these different forms of expression may compromise pain assessment [11]. Child-centered communication should therefore support symptom reporting, emotional needs, and meaningful participation in care.

In China, strengthening pediatric healthcare has become a national priority, and a national expert consensus published in 2024 specifically addressed pain management in children with hematologic diseases and tumors [12,13]. Nevertheless, children with hematologic malignancies remain exposed to pain associated with repeated procedures and treatment during hospitalization [7,14], and challenges in pediatric pain assessment and management persist [5,15,16]. These challenges may be particularly relevant to children from ethnolinguistic minority backgrounds, as language discordance can affect how children communicate pain and how healthcare professionals assess and respond to it [17,18]. Healthcare capacity in Tibet has expanded, including the ability to diagnose and treat childhood leukemia within the region [19]; however, linguistic accessibility remains important when children receive care in predominantly Mandarin-speaking settings. For Tibetan children who are less comfortable communicating in Mandarin, reliance on Mandarin verbal self-report alone may not fully capture their pain experiences, particularly when pain is communicated through verbal, behavioral, symbolic, or nonverbal forms [11,20].

Beyond linguistic communication, pain is a personal and lived experience shaped by biological, psychological, social, and relational contexts [21]. Religious or spiritual frameworks may also provide ways for some children and families to make sense of serious illness and suffering and to cope with distress [22,23]. However, the meaning and importance of such beliefs and practices vary among individuals and should not be inferred from Tibetan identity alone. Hospitalization and treatment may further disrupt schooling, peer relationships, and everyday routines [24], while unfamiliar clinical environments and repeated procedures may increase fear and distress. Parents and other family members may provide emotional security, companionship, and practical support during treatment [25,26]. These intersecting experiences highlight the importance of culturally responsive care that explores each child’s personal, relational, cultural, and spiritual context rather than presuming needs or responses on the basis of ethnicity.

Despite growing attention to pediatric cancer pain, evidence concerning how Tibetan children with hematologic malignancies perceive, communicate, and cope with pain within their particular sociocultural and linguistic contexts remains limited. Qualitative inquiry is well suited to addressing this gap because it enables in-depth exploration of children’s perspectives and lived experiences. Play-based interviewing may be particularly appropriate for eliciting children’s perspectives because it provides a developmentally sensitive and child-centered approach through which children can communicate experiences that may be difficult to articulate through conventional interviews alone [27,28].

Therefore, this descriptive phenomenological study used play-based interviews to explore the lived pain experiences of Tibetan children with hematologic malignancies and the sociocultural, linguistic, and relational contexts that may shape how pain is experienced, expressed, and managed. The findings are intended to inform linguistically accessible, culturally responsive, and child-centered pain assessment and nursing care.

## 2. Methods

### 2.1. Study design

This study employed a descriptive phenomenological design guided by Husserlian phenomenology. Descriptive phenomenology seeks to explore lived experiences as perceived and described by participants while minimizing the influence of researchers’ prior assumptions. Consistent with Husserl’s concept of epoché, the researchers used bracketing throughout data collection and analysis [29,30]. Before the interviews, the research team discussed and documented their prior clinical assumptions concerning children’s pain expression, coping behaviors, and Tibetan cultural practices in reflexive notes. During data collection and analysis, the researchers began with open-ended questions, used the prespecified topic prompts flexibly without suggesting preferred responses, and repeatedly returned to the children’s original accounts to minimize the imposition of external interpretations. Phenomenological reduction was applied to identify the meanings embedded in the children’s descriptions of their pain experiences. Data were collected through play-based, semi-structured, face-to-face interviews conducted from 4 February to 28 March 2026 in the pediatric hematology–oncology ward of Sichuan Provincial People’s Hospital, Sichuan Province, China. The study was reported in accordance with the Standards for Reporting Qualitative Research (SRQR) [31].

### 2.2. Participant recruitment

Purposive sampling was used to recruit Tibetan children with hematologic malignancies from the pediatric hematology–oncology ward of Sichuan Provincial People’s Hospital. This sampling strategy enabled the recruitment of participants who had directly experienced the phenomenon under investigation and could provide relevant experiential information.

The inclusion criteria were as follows: participants (1) were aged 4–12 years; (2) had a confirmed diagnosis of a hematologic malignancy; (3) were hospitalized for treatment at the time of recruitment; (4) had experienced pain related to the disease, treatment, or medical procedures; (5) were conscious and able to communicate at a basic level; and (6) spoke Tibetan as their first language or used Tibetan in daily communication, with or without Mandarin. Written informed consent was obtained from the parents or legal guardians, and verbal or written assent was obtained from the children when developmentally appropriate. Tibetan ethnicity was determined from the ethnicity recorded in the child’s medical record and confirmed by a parent or legal guardian; it was not inferred solely from language use.

Two researchers contacted the ward’s head nurse to introduce the study aims and procedures, after which ward staff assisted in identifying potentially eligible participants. The study was explained to the children and their caregivers using age-appropriate and culturally sensitive language. Children who met the eligibility criteria and agreed to participate were enrolled.

Recruitment proceeded alongside preliminary analysis until saturation was reached and confirmed. Saturation was operationally defined as the point at which consecutive interviews generated no new codes or substantively new meanings and did not require modification of the preliminary thematic structure. No new codes or meanings were identified after the 13th interview. Yi Chen and Yu Liu jointly made this saturation judgment and documented their discussion. Two additional interviews were conducted to confirm the stability of the thematic structure, resulting in a final sample of 15 children. Participant demographic and clinical characteristics are presented in Table 1.

**Table 1:** Demographic and Clinical Characteristics of Participants (N = 15).

Characteristics	Categories	n (%)
Age (years)	4–6	3 (20.0)
	7–9	7 (46.7)
	10–12	5 (33.3)
Gender	Male	8 (53.3)
	Female	7 (46.7)
Diagnosis	Acute lymphoblastic leukemia (ALL)	11 (73.3)
	Acute myeloid leukemia (AML)	4 (26.7)
Treatment phase	Maintenance	6 (40.0)
	Consolidation	4 (26.7)
	Relapse	3 (20.0)
	Induction	2 (13.3)
Residence	Urban	7 (46.7)
	Rural	8 (53.3)
Primary caregiver	Mother	7 (46.7)
	Father	3 (20.0)
	Grandparents	2 (13.3)
	Other relatives	3 (20.0)
Daily language	Tibetan	6 (40.0)
	Tibetan and Mandarin	9 (60.0)

### 2.3. Data collection

Data were collected from the children through play-based, semi-structured, face-to-face interviews. No separate or formal interviews were conducted with caregivers. When present, caregivers provided reassurance or necessary linguistic clarification only and were instructed not to answer on the child’s behalf. Caregivers were not treated as research participants or analytic data sources, and their statements were not coded, included in the thematic analysis, or used as supporting quotations. All themes and supporting quotations were derived from the children’s interview data.

The interview guide was used flexibly rather than as a fixed questionnaire. Interviews began with broad questions about the children’s pain and hospitalization experiences. The guide also included prespecified topic-specific prompts concerning emotional responses, spiritual or traditional coping practices, pain communication in Mandarin, and changes in school and peer relationships. These prompts were used as appropriate according to each child’s age, responses, and interview context. Follow-up probes were used to clarify or deepen the children’s accounts. The interview guide is presented in Table 2.

**Table 2:** Semi-structured interview guide.

No	Interview questions
1	Can you tell me about a time when you felt pain or discomfort in the hospital?
2	What did the pain feel like, and where did you feel it in your body? (You can show me by drawing or by using sand-tray materials or toys.)
3	How did you feel when the pain happened (for example, scared, sad, or angry)? What was the hardest part?
4	What do you think the pain means, or why do you think it happens? If you pray or use traditional ways of coping, do these help?
5	What do you usually do when you are in pain? What helps you feel better?
6	How do you let other people know that you are in pain? Is it easy or difficult to describe your pain in Mandarin?
7	What do you miss most during hospitalization, such as school or friends? Do you ever feel lonely here?

Note: The questions listed above constituted the semi-structured interview guide. Additional follow-up probes were used flexibly to clarify or deepen the children’s responses and were adapted to each child’s age, language ability, and preferred mode of communication.

Play-based interviewing followed a structured yet flexible procedure. At the beginning of each interview, the interviewer established rapport through brief informal interaction and introduced age-appropriate play materials, including drawing materials, toys, and sand-tray tools, to reduce anxiety and create a child-friendly environment. Children were invited to select materials according to their preferences, physical condition, language ability, and responsiveness.

Play activities were used as supportive communication aids rather than as therapeutic interventions. Children could indicate the location and nature of their pain through drawing, gestures, or by pointing to body parts on toys. Sand-tray materials were used to facilitate symbolic expression of pain experiences, emotions, and hospital-related situations. Examples of open-ended prompts were “Can you show me how it feels?” and “What is happening here?” The interview process was adapted to the children’s developmental needs. Younger children were encouraged to communicate through play, gestures, drawings, and brief verbal responses, whereas older children were invited to provide more detailed narratives with flexible prompts. Interviews lasted approximately 15–45 minutes and were conducted in the ward playroom. All interviews were audio-recorded with consent. Field notes documented verbal responses, nonverbal expressions, emotional responses, interactions with play materials, and relevant contextual information. Play-based expressions were interpreted together with the interview transcripts and field notes rather than analyzed as independent therapeutic or diagnostic data.

Five interviews were conducted entirely in Mandarin, whereas the remaining 10 involved Tibetan or a combination of Tibetan and Mandarin. During interviews involving Tibetan, real-time interpretation into Mandarin was provided by a hospital-based volunteer Tibetan–Mandarin interpreter who was a native Tibetan speaker, had received formal interpreter training, and had approximately three years of experience providing interpretation in clinical settings. The interpreter signed a study-specific confidentiality agreement before participating in the study. The initial Mandarin-language transcripts were jointly prepared by Yi Chen and the interpreter within 24 hours of each interview. For recordings containing Tibetan speech, the interpreter replayed the recordings and checked the Tibetan utterances against the corresponding Mandarin transcripts. Yi Chen and the interpreter discussed and resolved discrepancies and clarified culturally specific expressions before the transcripts were used for analysis.

The illustrative quotations selected for manuscript reporting were subsequently translated from Mandarin into English by Yi Chen, who had experience in reading and writing English-language nursing research. Yu Liu and Jinglan Zhang, both of whom had experience in reading and writing English-language nursing research, reviewed the translated quotations against the Mandarin transcripts, and any discrepancies were resolved through discussion. Because the English translations were prepared only for manuscript reporting and were not used for coding or thematic analysis, formal English-to-Mandarin back-translation was not performed.

### 2.4. Data analysis

Data collection and preliminary analysis were conducted concurrently. An adapted version of Colaizzi’s phenomenological method was used because it is consistent with descriptive phenomenology and emphasizes remaining close to participants’ descriptions when identifying the meanings of lived experiences [32]. The verified Mandarin transcripts of all 15 interviews served as the primary data for coding and thematic analysis. Yi Chen and Yu Liu independently read each transcript repeatedly to gain an overall understanding of the children’s pain experiences. They then identified significant statements and formulated meanings while remaining close to the children’s original accounts. The formulated meanings were compared across transcripts and organized into clusters of themes and subthemes reflecting shared experiential patterns. During theme development, linguistic and cultural influences were distinguished according to the experiential meaning and context of the children’s accounts but were recognized as potentially overlapping; relationships across themes were examined during interpretation rather than forcing the data into mutually exclusive categories. The researchers compared their coding and interpretations at each stage, and disagreements were resolved through discussion until consensus was reached. When linguistic or culturally specific meanings remained uncertain during coding and theme development, the analysts consulted the interpreter for clarification. The interpreter did not independently code transcripts or decide on the themes. An integrated description and fundamental structure of the children’s pain experiences were subsequently developed and checked against the verified Mandarin transcripts and field notes to ensure that the findings remained grounded in the data.

Participant validation, the final step of Colaizzi’s original method, was not conducted because the participants were young children undergoing treatment and additional follow-up was considered potentially burdensome. Therefore, the present study used an adapted rather than the complete seven-step Colaizzi method. NVivo 12 was used to organize and manage the data.

### 2.5. Trustworthiness

Trustworthiness was addressed through credibility, dependability, confirmability, and transferability. Credibility was supported through investigator triangulation, iterative research-team debriefing, comparison of the thematic findings with the interview accounts and field notes, and linguistic verification. For interviews involving Tibetan, the interpreter checked the Tibetan content in the audio recordings against the corresponding Mandarin transcripts, and Yu Liu and Jinglan Zhang reviewed the English translations used for manuscript reporting against the Mandarin transcripts. These strategies were used as alternatives to participant validation (member checking). Dependability was supported by maintaining an audit trail that included records of interview procedures, coding decisions, theme development, and revisions to the thematic structure. Confirmability was enhanced through reflexive notes and team discussions examining how the researchers’ clinical backgrounds and prior assumptions might influence data collection and interpretation. Transferability was supported through detailed descriptions of the study setting, participant characteristics, sampling process, data collection procedures, and children’s pain experiences, enabling readers to assess the relevance of the findings to other contexts.

### 2.6. Researcher Reflexivity

The child interviews were conducted by the first author, Yi Chen, a postgraduate nursing student who had received systematic training in qualitative research, and two pediatric hematology–oncology nurses, Juan Zhou and Huarong Pu, who had received qualitative interviewing training and each had approximately 10 years of clinical experience in pediatric hematology–oncology. Juan Zhou and Huarong Pu had established clinical relationships with the children through their nursing roles, whereas Yi Chen was not a clinical nurse on the study ward and was not involved in the participants’ clinical care. The nurses’ familiarity with the children may have facilitated rapport; however, it may also have encouraged socially desirable or acquiescent responses.

None of the interviewers were Tibetan or fluent in Tibetan. A hospital-based volunteer Tibetan–Mandarin interpreter with experience providing language support in clinical settings therefore provided real-time interpretation when Tibetan was used, as described in the Data Collection section. The research team acknowledged that the interviewers’ clinical experience, prior relationships with the children, and cultural–linguistic positions could influence data collection and interpretation. To minimize these influences, the interviewers began with broad, open-ended questions, reminded the children that participation was voluntary and would not affect their care, and encouraged them to describe their experiences in their own ways. Data analysis was conducted independently by Yi Chen and Yu Liu. Yu Liu did not participate in the interviews and was a nurse manager and a postgraduate nursing student with approximately 20 years of oncology nursing experience. She had received training in qualitative research and had prior experience in qualitative data analysis. She was not Tibetan and was not fluent in Tibetan. During the analytic process, independent coding was followed by reflexive discussions between Yi Chen and Yu Liu about how their professional backgrounds, cultural–linguistic positions, and prior assumptions might influence coding and interpretation. Throughout data collection and analysis, the researchers maintained reflexive notes and repeatedly checked their interpretations against the original transcripts and field notes.

### 2.7. Ethical Considerations

This study was approved by the Ethics Committee of Sichuan Provincial People’s Hospital (Ethics Approval No. 2026−81) and was conducted in accordance with the Declaration of Helsinki. Written informed consent was obtained from the parents or legal guardians of all participating children, and verbal or written assent was obtained from the children according to their age and developmental capacity. Because two of the interviewers were nurses familiar with the children and involved in their clinical care, the children and their guardians were explicitly informed that participation was voluntary and that declining or withdrawing from the study would not affect the children’s treatment, nursing care, or relationships with healthcare professionals. Children could decline to answer any question and pause or end the interview at any time. Interviews were scheduled according to each child’s physical condition and were paused or discontinued if the child showed signs of fatigue, distress, pain, or discomfort. Audio recordings, transcripts, and field notes were de-identified, stored on password-protected devices, and accessible only to the research team.

## 3. Results

A total of 15 Tibetan children with hematologic malignancies participated in this study. Four themes and nine subthemes were identified from the children’s interview data: (1) cultural and spiritual interpretations of pain; (2) linguistic and communicative constraints in pain expression; (3) family-centered dimensions of pain experience; and (4) hospitalization-related disruptions to everyday social life. These themes reflect how the children described and responded to pain in relation to cultural and spiritual meanings, language and communication, family relationships, and disruptions to their everyday social lives. The resulting thematic structure is presented in Table 3.

**Table 3:** Themes and Subthemes of Tibetan Children’s Pain Experiences.

Themes	Subthemes
1. Cultural and spiritual interpretations of pain	1.1 Silent endurance of suffering
	1.2 Religious and traditional coping practices
2. Linguistic and communicative constraints in pain expression	2.1 Limited Mandarin vocabulary for pain
	2.2 Indirect expression of discomfort
3. Family-centered dimensions of pain experience	3.1 Emotional security through family presence
	3.2 Relational continuity through family accompaniment
	3.3 Reliance on familiar family-based comfort practices
4. Hospitalization-related disruptions to everyday social life	4.1 Disrupted schooling and peer contact
	4.2 Concerns about illness disclosure and social isolation

Across the four themes, the fundamental structure of the children’s lived experience was that pain was not an isolated bodily sensation; it was culturally and spiritually meaningful, difficult to render communicable, mediated through family relationships, and intertwined with disruption to ordinary childhood. These dimensions were interrelated: how children endured or communicated pain was shaped by the meanings they assigned to it, the linguistic resources available to them, the presence of family, and their separation from school and peers.

### Theme 1. Cultural and spiritual interpretations of pain

#### Subtheme 1.1 Silent endurance of suffering.

Some participants described attempts to tolerate pain quietly rather than immediately reporting it. Three boys viewed openly expressing pain as inconsistent with being strong and described efforts to remain brave during painful experiences. For these boys, remaining silent appeared to represent more than an absence of pain reporting; it was associated with maintaining a sense of themselves as strong and brave while experiencing pain.

One child said, “We think boys shouldn’t be too delicate. If we feel pain, we just bear it ourselves. If we say it out loud, others will think we are not strong.” (N2)

Another child said, “I am a little man. I can’t cry. I have to be brave when it hurts.” (N5)

A third child said, “My head hurts and it feels really uncomfortable, but I have to overcome it like a warrior.” (N9)

#### Subtheme 1.2 Religious and traditional coping practices.

In response to questions about ways of understanding or coping with pain, some children described prayer, scripture recitation, temple visits, or traditional Tibetan remedies. These practices were described alongside hospital treatment as part of their everyday ways of facing illness and discomfort. In these accounts, spiritual and traditional practices appeared to help some children make sense of pain and experience it as less frightening or more endurable.

One child said, “When it hurts, I recite scriptures. I feel a bit better, like the lama is with me, and the pain doesn’t feel so scary.” (N1)

Another child said, *“*My grandma spins the prayer wheel for me and teaches me to chant. That makes me feel less afraid.*”* (N4)

One child also said, “We also go to the temple to pray for blessings.*”* (N6)

Another child said, “I think this pain is a spiritual test. If I can get through it, something good may happen later.” (N11)

Regarding traditional medicine, one child said, “I know Tibetan medicine will protect me.” (N10) Another child said, “My mom gives me Tibetan medicine. It helps reduce the pain and makes my body feel more comfortable.” (N7)

### Theme 2. Linguistic and communicative constraints in pain expression

Although indirect pain expression was grouped under this theme because it was frequently described in situations where children had difficulty verbalizing pain, linguistic and cultural influences were not treated as mutually exclusive. For some participants, silence or indirect expression appeared to intersect with the tendency to endure discomfort described in Theme 1.

#### Subtheme 2.1 Limited Mandarin vocabulary for pain.

Several children described difficulty finding words in Mandarin to express pain. When communicating with healthcare workers, they often relied on simple or general terms rather than detailed descriptions. Some children also referred to body parts, gestures, or facial expressions when verbal expression was limited. The accounts therefore revealed a gap between pain as inwardly understood and pain as expressible in a Mandarin-dominant clinical encounter.

One child said, “I don’t know how to say it in Chinese. I just tell them it hurts here.” (N2)

Another child said, “I can say it in Tibetan, but in Mandarin I don’t know the words, so I just say uncomfortable.” (N14)

A third child said, “I don’t know how to explain my pain clearly in Mandarin. I understand it in my heart, but I can’t say it well, so the doctor may not fully understand me.” (N7)

#### Subtheme 2.2 Indirect expression of discomfort.

Some children did not describe pain directly. Instead, they said they felt tired, wanted to sleep, or felt uncomfortable. Some children also remained quiet when the pain became stronger. Silence and the general term “uncomfortable” functioned as indirect forms of pain communication, whereas wanting to sleep represented an indirect way of responding to or coping with pain.

One child said, “When it hurts, I just want to sleep. If I sleep, it feels better.” (N10)

Another child said, “I don’t usually say that I am in pain. I just say that I feel uncomfortable.” (N6)

A third child explained, “When the pain gets worse, I don’t want to talk. I just stay quiet.” (N13)

### Theme 3. Family-centered dimensions of pain experience

Whereas Theme 1 concerned the meanings children attributed to spiritual and traditional practices, Theme 3 concerned how pain was experienced and managed through family relationships.

#### Subtheme 3.1 Emotional security through family presence.

For several children, family presence represented more than practical companionship during painful episodes; it provided a sense of emotional security and familiarity within an unfamiliar hospital environment. Children described feeling less frightened or distressed when a parent, particularly their mother, remained physically close. Holding hands, seeing a familiar family member at the bedside, or simply knowing that a parent was nearby appeared to make pain feel more manageable. Thus, family presence was described by children as an important part of their experience of pain and coping during hospitalization.

One child said, “When my mom holds my hand, I feel less scared, even if it still hurts.” (N14)

Another child said, “If my mom is here, I don’t feel as bad. If she leaves, I feel more uncomfortable.” (N6)

A third child said, “My mom is always with me. When I see her, I feel less pain.” (N3)

#### Subtheme 3.2 Relational continuity through family accompaniment.

Children described being accompanied by different family members during hospitalization. The presence of mothers, fathers, aunts, and other relatives helped maintain familiar relationships and a sense of connection with life outside the hospital. For some children, accompaniment by different family members carried a sense of home into the hospital and preserved relational continuity and stability across repeated admissions.

One child said, “I have two aunts. Last time one aunt brought me here from Tibet, and this time my mom brought me.” (N13)

Another child said, “My parents both come to stay with me. Someone from my family is always here.” (N12)

A third child said, “Although I am not at home, my family is still with me.” (N10)

#### Subtheme 3.3 Reliance on familiar family-based comfort practices.

Children’s accounts indicated that family responses to pain included immediately available comfort measures, such as warmth, massage, and familiar remedies routinely used at home. For the children, the meaning of these practices appeared to lie not only in the action itself but also in receiving care from a trusted family member.

One child said, “When I feel uncomfortable, my mom puts something warm on my stomach and applies Tibetan medicine.” (N10)

Another child said, “When my mother massages me, I feel more comfortable.” (N11)

### Theme 4. Hospitalization-related disruptions to everyday social life

When discussing hospitalization-related changes, children described disrupted schooling, reduced peer contact, and concerns about illness disclosure. This theme reflected the social context in which pain and hospitalization were experienced, rather than consequences attributable to pain alone.

#### Subtheme 4.1 Disrupted schooling and peer contact.

Many children mentioned school and classmates when describing their lives during treatment. They talked about missing daily activities such as studying, playing, and spending time with friends. Some children referred to these absences when talking about discomfort and hospitalization. These accounts reflected a temporary separation from ordinary childhood roles and routines, as well as disruption to anticipated transitions for some children.

One child said, “I really want to go back to school. I miss my friends and playing together.” (N1)

Another child said, “When I see other kids going to school outside, I feel a bit sad.” (N3)

A third child said, “I’m supposed to start junior high. I want to go back to school. I miss my classmates.” (N2)

#### Subtheme 4.2 Concerns about illness disclosure and social isolation.

Some children experienced social isolation during hospitalization, while others expressed concerns about disclosing their illness to peers. Together, these accounts revealed a tension between wanting peer connection and keeping illness private.

One child said, “I don’t have friends here. I just stay with my mom.” (N6)

Another child explained, “I am afraid that my classmates will know about my illness, so I usually do not tell them.” (N14)

## 4. Discussion

This study explored the pain experiences of Tibetan children with hematologic malignancies through play-based interviews. Previous pediatric oncology studies have examined pain outcomes [33], communication [34], spirituality [35], and parental roles in pain management [36] largely as separate topics. Our findings bring these dimensions together from children’s own accounts and show that pain was experienced within interrelated cultural, linguistic, relational, and everyday-life contexts. From a phenomenological perspective, pain was therefore more than a biomedical symptom: it was situated within sociocultural, developmental, and relational contexts. The contribution lies in showing how these contexts intersect rather than treating culture, language, and family as background characteristics. This supports culturally responsive, child-centered pain assessment for ethnolinguistic minority children.

### 4.1. Cultural and spiritual meanings of pain: recognizing silent endurance without stereotyping

Some children interpreted pain through cultural and spiritual meanings rather than solely as a physical symptom. Several described enduring discomfort quietly, and boys in this sample connected silence with being strong, brave, or a “little man.” Silent endurance may therefore intersect with age- and gender-related expectations, personal coping, and family influences; it should not be interpreted as low pain intensity or effective pain control. Prayer, scripture recitation, spiritual testing, and Tibetan remedies were described as familiar coping resources, consistent with evidence that spirituality may provide meaning and comfort for children with cancer and their families [23,35]. Among Tibetan Buddhist believers in earthquake-affected Yushu, concepts such as karma, prayer, chanting, and religious relationships supported meaning-making and resilience [37]. However, children in the present study did not explicitly describe pain in terms of karma or destiny, so these concepts were not treated as findings. Their accounts instead suggested that spiritual practices placed pain within a meaningful narrative; describing pain as “a spiritual test” (N11), for example, linked suffering with hope. Spiritual meaning-making did not eliminate pain but appeared to make it less frightening and helped some children continue coping with an experience they could not fully control. Quiet endurance may nevertheless delay disclosure and lead clinicians to underestimate pain. These findings do not imply that Tibetan children universally accept pain. Assessment should ask each child what silence, prayer, traditional practices, and spiritual beliefs mean to them while prioritizing the child’s report and clinical needs.

### 4.2. Linguistic and communicative constraints: moving beyond literal translation

Linguistic constraints shaped pain communication. Several children could describe pain in Tibetan but struggled to express its location, quality, intensity, or change in Mandarin, consistent with evidence that linguistic minoritization can contribute to inequities in pediatric pain assessment [11,17,18]. The barrier extended beyond vocabulary: children had to render an embodied sensation understood in Tibetan into Mandarin terms recognizable in a Mandarin-dominant clinical encounter. “Uncomfortable,” pointing, silence, or withdrawal could therefore signal pain, whereas wanting to sleep represented a response or coping strategy. Because indirect expression intersected with silent endurance, linguistic and cultural processes could compound one another. Assessment should consider whether the child can describe pain in clinically useful terms and correct adults’ interpretations, not merely whether the child speaks Mandarin. Developmentally appropriate questions and sufficient response time may facilitate expression, while support from a trained Tibetan–Mandarin interpreter and confirmation of meaning may reduce misunderstanding [38]. Visual tools, body diagrams, gestures, drawing, toys, and play-based prompts offer alternative channels when verbal fluency is limited [34]. Caregivers may clarify familiar words or behaviors, but professionals should continue to address the child directly and avoid allowing adult interpretation to replace the child’s account [39].

### 4.3. Family-centered pain experiences: supporting caregivers as relational and communication partners

For several children, family presence provided emotional security, familiarity, and relational continuity during repeated hospitalizations. Consistent with pediatric oncology literature [36,40], family members bridged home and hospital, reduced fear, and supported familiar comfort practices such as hand-holding, warmth, massage, and Tibetan remedies. Family participation requires structured guidance. Nurses can teach caregivers to recognize the child’s usual verbal, behavioral, and nonverbal pain signals; report pain location, timing, triggers, changes, and responses to comfort measures; and notify staff when the child becomes unusually quiet or withdrawn [41]. Caregivers should report Tibetan remedies and use comfort practices only after their safety and treatment compatibility have been reviewed [42]; they may support interpretation without answering for the child [38]. A brief bedside pain plan documenting preferred words, nonverbal signals, helpful strategies, and family roles may integrate relational support with professional assessment.

### 4.4. Hospitalization-related disruptions to everyday social life: preserving ordinary childhood and peer connection

Children described disrupted schooling, play, and peer contact during hospitalization, together with concerns about disclosing their illness. These experiences distanced them from ordinary childhood. Cancer symptoms can interfere with daily functioning [4,6], while social connection may benefit hospitalized children [24]. Concerns that peers might learn about the illness also suggest uncertainty about stigma, privacy, and belonging [43]. These experiences formed the context in which pain and hospitalization were lived but do not establish that pain alone caused school interruption or isolation. Pain care focused only on symptom intensity may overlook losses in routine, identity, and participation that shape children’s distress. Nursing teams can assess school and peer concerns, facilitate age-appropriate learning or safe virtual contact when feasible, and discuss illness disclosure according to the child’s preferences rather than assuming that peer contact is always desired.

### 4.5. Methodological contribution of play-based interviewing

The use of age-appropriate play materials was a methodological strength of this study. The materials were not used as therapeutic or diagnostic tools; rather, they provided supportive channels through which children could locate pain on a toy, communicate through gestures or drawing, and describe difficult experiences in brief or symbolic narratives. Building on previous work on narrative and play-based interviewing [27,28], this approach may be particularly valuable when developmental stage and language discordance coexist. By reducing reliance on fluent Mandarin and extended verbal explanation, it may have helped preserve access to the child’s own perspective. Nevertheless, play-based expressions were interpreted together with interview transcripts and field notes and should not be treated as stand-alone evidence of pain or cultural meaning.

## 5. Limitations

This study has several limitations. First, participants were recruited from a single tertiary hospital and included only hospitalized Tibetan children with hematologic malignancies, which may limit the transferability of the findings to other populations and care settings. Second, each child was interviewed only once; therefore, changes in pain experiences across different treatment stages were not examined. Although play-based interviewing facilitated children’s expression, some pain experiences may still have been difficult to articulate, particularly for younger children and those with limited verbal abilities. The interview guide included topic-specific prompts concerning spiritual or traditional coping practices, pain communication in Mandarin, and school and peer relationships. These prompts may have influenced the topics discussed and the emphasis placed on particular experiences. No separate or formal caregiver interviews were conducted, and caregivers were not treated as analytic data sources; however, their presence during the interviews may have influenced how some children described their experiences, despite being instructed not to answer on the child’s behalf. Because two interviewers were nurses involved in the children’s clinical care and had pre-existing clinical relationships with them, some children may have provided socially desirable or acquiescent responses, despite assurances that participation was voluntary and efforts to encourage children to respond in their own words. Although the interpreter checked the Tibetan content in the audio recordings against the corresponding Mandarin transcripts and Yu Liu and Jinglan Zhang reviewed the English translations of illustrative quotations against the Mandarin transcripts, the multilingual translation process may still have introduced subtle shifts in linguistic or culturally specific meanings. Participant validation was not conducted because of the children’s young age and ongoing treatment burden; consequently, the final thematic structure was not directly confirmed by participants. Because the interviewers and analysts were not Tibetan and were not fluent in Tibetan, their cultural-linguistic and professional positions may have influenced the phenomenological interpretation despite reflexive discussions and repeated comparison with the original data. Finally, the cultural interpretations identified in this study should be understood as the individual and context-specific experiences of the participants rather than as representative of all Tibetan children with hematologic malignancies.

## 6. Implications for nursing practice and future research

The findings suggest that pain assessment for Tibetan children with hematologic malignancies may need to consider not only pain intensity but also the cultural, linguistic, relational, and social contexts in which pain is understood and expressed. Simple language, visual supports, sufficient response time, support from a trained Tibetan–Mandarin interpreter, and developmentally appropriate play-based communication may facilitate children’s expression and reduce misunderstanding. Caregivers may provide emotional security and help clarify familiar verbal or behavioral cues, provided that the child remains the primary source of information and caregivers do not answer on the child’s behalf. Nursing care may also attend to disrupted schooling, peer connection, and children’s preferences regarding illness disclosure.

Future multisite and longitudinal studies could include more diverse pediatric populations from Tibetan and other ethnolinguistic minority communities and examine how cultural meanings, language, family relationships, and social experiences interact across treatment stages. Intervention studies could evaluate the feasibility and effectiveness of trained language support, play-based pain communication, and structured family education. Such research should continue to treat culture as individual and context specific rather than as a fixed explanation for all children within an ethnic group.

## 7. Conclusions

The pain experiences of Tibetan children with hematologic malignancies were embedded in cultural and spiritual meanings, linguistic and communicative constraints, family relationships, and hospitalization-related disruptions to everyday social life. Play-based interviewing provided developmentally appropriate avenues for expression when extended verbal communication was difficult. These findings contribute to understanding pediatric cancer pain in an ethnolinguistic minority context and may inform culturally responsive, child-centered pain assessment and nursing care.
